# Sleep–wake regulation in preterm and term infants

**DOI:** 10.1093/sleep/zsaa148

**Published:** 2020-08-08

**Authors:** Anastasis Georgoulas, Laura Jones, Maria Pureza Laudiano-Dray, Judith Meek, Lorenzo Fabrizi, Kimberley Whitehead

**Affiliations:** 1 Research IT Services, University College London, London, United Kingdom; 2 Department of Neuroscience, Physiology and Pharmacology, University College London, London, United Kingdom; 3 Elizabeth Garrett Anderson Wing, University College London Hospitals, London, United Kingdom

**Keywords:** neonate, premature, pain

## Abstract

**Study Objectives:**

In adults, wakefulness can be markedly prolonged at the expense of sleep, e.g. to stay vigilant in the presence of a stressor. These extra-long wake bouts result in a heavy-tailed distribution (highly right-skewed) of wake but not sleep durations. In infants, the relative importance of wakefulness and sleep are reversed, as sleep is necessary for brain maturation. Here, we tested whether these developmental pressures are associated with the unique regulation of sleep–wake states.

**Methods:**

In 175 infants of 28–40 weeks postmenstrual age (PMA), we monitored sleep–wake states using electroencephalography and behavior. We constructed survival models of sleep–wake bout durations and the effect of PMA and other factors, including stress (salivary cortisol), and examined whether sleep is resilient to nociceptive perturbations (a clinically necessary heel lance).

**Results:**

Wake durations followed a heavy-tailed distribution as in adults and lengthened with PMA and stress. However, differently from adults, active sleep durations also had a heavy-tailed distribution, and with PMA, these shortened and became vulnerable to nociception-associated awakenings.

**Conclusions:**

Sleep bouts are differently regulated in infants, with especially long active sleep durations that could consolidate this state’s maturational functions. Curtailment of sleep by stress and nociception may be disadvantageous, especially for preterm infants given the limited value of wakefulness at this age. This could be addressed by environmental interventions in the future.

**Statement of Significance**

Sleep dominates neonatal life and is known in animals to be necessary for normal brain development. However, how sleep–wake states are regulated in human infants is unknown. To resolve this, we monitored sleep–wake cycling in 175 preterm infants spanning the 12 weeks leading up to the average time of birth. The results show that sleep is differently regulated in human neonates to adults, leading to prolonged rapid eye movement sleep bouts, and that clinically necessary painful procedures and physiological stress perturb this intrinsic regulation. This study identifies tractable factors that could be targeted to protect sleep in the preterm population, which we discuss together with a conceptual sleep–wake regulatory model consistent with basic neurobiology.

## Introduction

In adults, positive feedback mechanisms within brainstem circuitry have likely evolved to allow wakefulness to be extensively prolonged at the expense of sleep when necessary, e.g. to stay vigilant in the presence of a fitness-reducing stressor such as a predator [1]. These extra-long wake bouts result in a heavy-tailed distribution (i.e. highly right-skewed) of wake durations but not sleep durations. Instead, sleep durations are comparatively capped in length [2].

In early mammalian life, the relative importance of wakefulness and sleep are reversed. In neonatal animal models, active sleep in particular (precursor to rapid eye movement [REM] sleep) is more efficient than wakefulness at supporting neural activity-dependent sensorimotor development [3–5]. Consequently, sleep deprivation and suppression of sleep behavioral patterns can impair cortical activity levels and synaptic plasticity [6–8]. In human infants, sleep-specific motor activity evokes somatotopic cortical activity [9, 10], and learning can take place during sleep [11]. Meanwhile, wakefulness may confer relatively little advantage, especially in preterm infants who are too young to demand feed (they are fed via a nasogastric tube, which can happen while asleep) and have little capacity to “fight or flight” to a stressor. Here, we tested whether these ontogenetic pressures are associated with unique regulation of sleep–wake states and the influence of demographic and environmental factors relevant to this population, including age, stress, and sensory perturbations.

## Methods

### Participants

In total, 175 infants spanning the 12 weeks leading up to the average time of birth (40 weeks postmenstrual age [PMA]; [12]) were recruited from the neonatal and postnatal wards at the Elizabeth Garrett Anderson wing of University College London Hospitals between July 2015 and October 2019 for research examination (Table 1). No neonates were acutely unwell, mechanically ventilated, or had received sedative medications in the 24 hours prior to the study. Two out of 175 infants were receiving a weaning regime of oral morphine.

**Table 1.** Demographics of 175 participants

Sex	89 female: 86 male
Gestational age (weeks + days)	23 + 2 to 40 + 1 (median 34 + 3)
Postnatal age (days)	0.5 to 96
PMA (weeks + days)*	28 + 2 to 40 + 1 (median 35 + 4)
Subgroups (weeks + days)	Very preterm (28 + 2 to 31 + 6), *n* = 29 Moderately preterm (32 + 0 to 33 + 6), *n* = 22 Late preterm (34 + 0 to 36 + 6), *n* = 66 Full-term (37 + 0 to 40 + 1), *n* = 58
Ward location at the time of study^†^	112 neonatal ward; 63 postnatal ward

*PMA = gestational age + postnatal age.

^†^Infants on the neonatal ward require close nursing and medical care; infants on the postnatal ward are cared for by their parents.

Ethical approval was obtained from the NHS Research Ethics Committee, and informed written parental consent was obtained prior to each study. Additional written parental consent was obtained to publish a photograph of one infant.

### Sleep–wake state assessment

**Figure 1.**
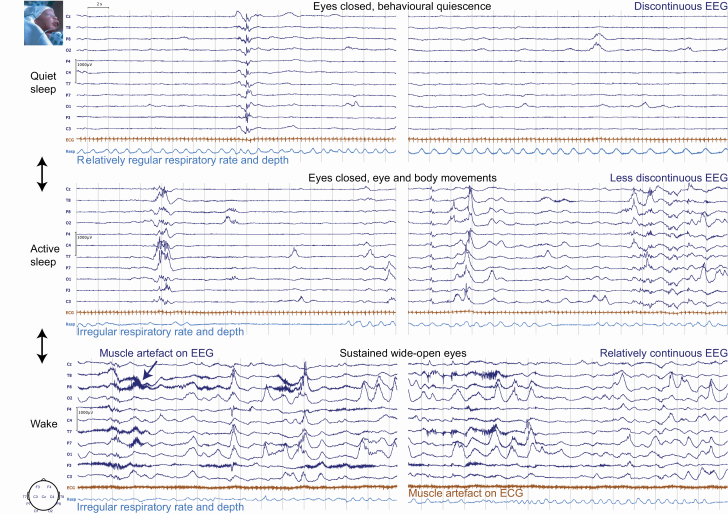
Two examples each of epochs indicating quiet sleep, active sleep, and wakefulness in the same infant 29 + 3 weeks + days PMA. Two-headed arrows depict the flow of state transitions (see Table 2).

Sleep–wake states were defined using electroencephalography (EEG) (mean 17 recording electrodes [range 2–19]), heart rate, respiratory, and cot side behavioral monitoring. Recordings had a median length per subject of 57 minutes (interquartile range: 44–70 minutes) and commenced between 07:00 am and 19:00 pm. All infants were offered individualized, developmentally appropriate comfort measures during electrode placement as and when required (e.g. swaddling if they became unsettled) [13]. Recordings were acquired and then manually scored by the same clinical scientist (K.W.) as wakefulness, active sleep, transitional sleep, or quiet sleep in 30-second epochs (Figure 1), according to the criteria of the American Academy of Sleep Medicine for infants [14] (using Analyzer 2’s Sleep Scoring Solution [Brain Products]). In order to score a state transition, the minimum length of the new sleep–wake state was 1 minute, as in previous neonatal studies [15–19].

**Table 2.** Sleep–wake bouts

State	No. of bout onsets captured	No. when bout onset *and* offset captured	State that the bout offset into
Active sleep	183 (from 135 infants 28–40 weeks PMA)	75/183	Quiet sleep 49/75 Wakefulness 26/75
Quiet sleep	157 (from 132 infants 28–40 weeks PMA)	118/157	Active sleep 117/118 Wakefulness 1/118
Wakefulness	43 (from 40 infants 29–40 weeks PMA)	29/43	Active sleep in 29/29

PMA = gestational age + postnatal age.

Wakefulness was defined by continuously or almost continuously wide-open eyes, or closed or obscured eyes if crying or feeding, respectively, high muscle tone, and profuse movements. Active sleep was defined by closed eyes with intermittent REMs, isolated facial and body movements, brief vocalizations, largely irregular breathing, and relatively continuous EEG compared with quiet sleep. Quiet sleep was defined by closed eyes, almost complete behavioral quiescence, and relatively regular and/or deep breathing and discontinuous EEG compared with active sleep and wakefulness. Transitional sleep was scored when eyes were closed and there was no crying, but other characteristics provided equal support for active or quiet sleep.

### Assessing the influence of sensory perturbations and physiological stress

A subset of 102 infants received at least one clinically necessary heel lance during the recording and 135 infants received at least one non-noxious (control) mechanical somatosensory stimulus (sham heel lance, see 04:05 into video here [20]) or tap (see videos here [21, 22]). The infants who received a lance or non-noxious stimulus did not differ in PMA from infants who did not receive these (Mann–Whitney *U* test: lance *p* = .775, non-noxious *p* = .215). In 55 of the infants who received a lance during the recording, a salivary cortisol value was available (Supplementary Figure S1). Cortisol samples were collected before, during, and at the end of the recording (methodology described in [23]). Cortisol concentrations did not significantly differ across these time points (*p* = .854; Friedman’s two-way analysis of variance by ranks, *n* = 19 participants for whom all three samples were available), so we used the average of those values available per subject as a measure of physiological stress throughout the test period. (Cortisol production does not yet follow a circadian rhythm in neonates [24].) In order to assess interactions between the sensory environment and sleep–wake state, we annotated the occurrence of nociceptive and non-noxious stimuli in parallel with the sleep–wake scores as well as the infant’s held status (held/unheld by a caregiver).

### Analysis: distributions of sleep–wake bout durations

**Figure 2.**
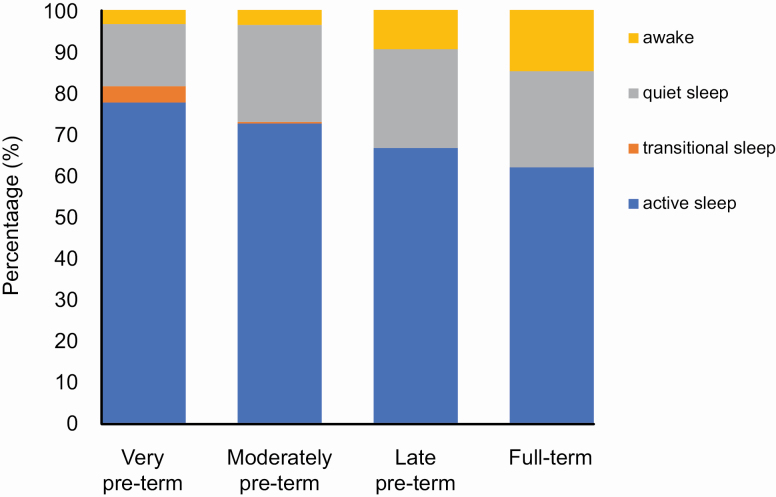
The mean proportion of time spent in wakefulness, quiet sleep, transitional sleep, and active sleep in infants of very preterm, moderately preterm, late preterm, and full-term PMA (Table 1).

We first calculated the mean percentage of each sleep–wake state during the recording in very, moderately, and late preterm infants, and full-term infants (Table 1; Figure 2) [12, 25]. However, to investigate developmental changes in sleep–wake regulation, it is necessary to model bout durations. Bout onsets were identified using custom-written software code. To characterize the distributions of wakefulness, active sleep, and quiet sleep bout durations, we constructed parametric Accelerated Failure Time “survival” models using R package flexsurvreg [26]. Survival models take account of “censored” observations, i.e. bout durations contribute to the model even if bout offset was not captured. This is important because extra-long bouts may not offset during the recording but are of particular interest. The advantage of *parametric* survival models is that bout durations can be shown to follow statistical distributions commonly found throughout biology, for which there is a large literature on generative mechanisms [27].

Following review of the statistical distributions used to characterize sleep–wake bout durations in the literature [28, 29], bout durations were tested against three distinct alternative distributions of increasing right-skew: (1) light-tailed (right tail may be long but is lightly skewed relative to an exponential distribution): Weibull distribution with shape parameter >1, (2) exponential, or (3) heavy-tailed (right tail is very long and heavily skewed relative to an exponential distribution): lognormal was the heavy-tailed distribution tested; a power law was ruled out using R package poweRlaw [30, 31]. Which distribution fits the data provides insight into state regulation. An exponential distribution indicates that transitions out of the bout are random [32]; sleep bout durations in healthy adults fit an exponential distribution [33]. A bout duration distribution that is either lighter- or heavier-tailed than an exponential distribution indicates that transitions out of the bout cannot be random. For example, sleep bouts in adults with sleep-disordered breathing fit a light-tailed distribution (very little probability of extra-long sleep bouts), which has been interpreted as a bias toward the opposing state—i.e. wakefulness—secondary to the wakefulness-inducing apneas/hypopneas [28]. On the other hand, *wake* bouts in adults fit a *heavy*-tailed distribution (higher probability of extra-long wake bouts) [33], which can be interpreted as *more* state stability, e.g. via positive feedback mechanisms, which make long-lasting bouts even more likely to endure.

The model representing the distribution of the durations of each sleep–wake state was selected as the one which minimized Akaike’s information criterion (AIC). Using this criterion addresses the risk of model overfitting, because it penalizes models with a higher number of parameters. After establishing which model distribution fit the bout durations of each sleep–wake state, we modeled changes in the parameters of those fits according to demographic and environmental variables. We selected PMA as the most likely explanatory variable, based upon the existing literature [18, 34–36]. We selected postnatal age, lower vs. higher risk of adverse neurodevelopment (see Supplementary Information), a preceding sensory perturbation, physiological stress, and position of the bout within the sleep–wake cycle (i.e. which transition occurred at bout offset) to additionally enter into the model as potential secondary explanatory variables, according to previous work [16, 37–42]. The first three variables, which were available for all data points, were entered into the overall models. For the latter variables that were only available for a subset of data points, we constructed separate models for these smaller datasets. Variables were defined as improving model fit if they reduced AIC, and the internal validity of model fits was evaluated based upon a graphical comparison between empirical Kaplan–Meier survival curves (derived from the data) and fitted survival curves (generated from the models). To provide a visual representation of continuous variables that influenced sleep–wake bout durations, we generated survival curves in which the variable was split into two groups (e.g. lower and higher PMA). Additional analysis was carried out using SPSS version 26. The statistical significance threshold was set to 0.05 for all tests.

## Results

The mean proportion of time spent in active sleep and transitional sleep decreased with PMA, alongside an increase in the proportion of time spent in quiet sleep and awake: in particular, the proportion of wakefulness increased from just 3% in very preterm infants to 15% in full-term infants (Figure 2).

### Sleep and wake bout durations were differently distributed

Characterizing the relative percentage of each sleep–wake state cannot capture their time courses, which differed markedly between infants (Supplementary Figure. S2). (For example, 50% prevalence each of active and quiet sleep during a 1-hour period could reflect consecutive short 1-minute bouts or two consolidated 30-minute bouts.) Therefore, we next analyzed data at the level of the onset and duration of each sleep–wake bout [43] (Table 2).

**Figure 3.**
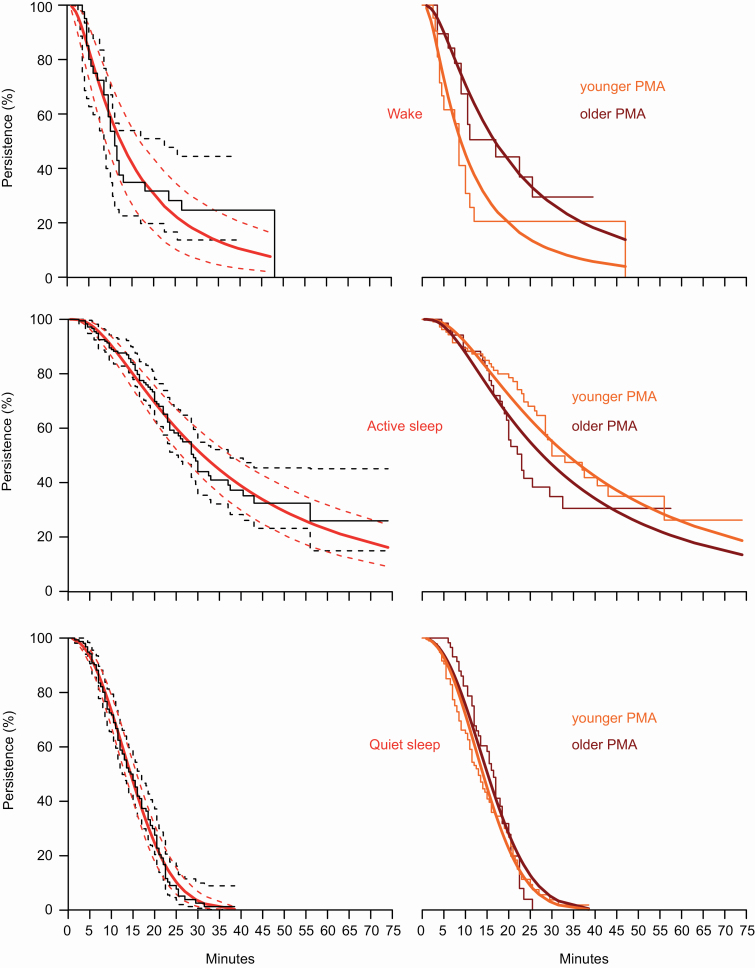
Survival curves for persistence of wakefulness, active sleep, and quiet sleep. Left panel: fitted survival curves (red lines) and confidence intervals (red-dashed lines), alongside empirical Kaplan–Meier survival curves (black lines) and confidence intervals (black-dashed lines). Curves are drawn with the covariate of PMA set to its mean values in the data. Right panel: fitted survival curves for younger infants (<36 weeks PMA, orange lines) and older infants (≥36 weeks PMA, crimson lines). Confidence intervals omitted here for clarity.

Wake and active sleep bout durations were most consistent with a heavy-tailed distribution: the exit rate from the state eventually plateaued (at approximately 10 and 30 minutes, respectively), resulting in a small number of extra-long bouts (Table 3; Figure 3 left panel). On the other hand, quiet sleep bout durations were most consistent with a light-tailed distribution (i.e. very little probability of extra-long bouts) (Table 3; Figure 3 left panel).

**Table 3.** Goodness of fit of models of sleep–wake bout durations

	Model	AIC	K	Delta	Estimates
Wakefulness	**Lognormal**	**264.629**	**2**	**0**	Mean log 3.187 (95% CI: 2.860 to 3.514), SE: 0.167 SD log 0.996 (95% CI: 0.762 to 1.302), SE: 0.136
	Weibull	273.282	2	8.6531	
	Exponential	271.608	1	6.9792	
Active sleep	**Lognormal**	**821.681**	**2**	**0**	Mean log 4.137 (95% CI: 3.959 to 4.314), SE: 0.091 SD log 0.891 (95% CI: 0.755 to 1.051), SE: 0.075
	Weibull	827.129	2	5.4476	
	Exponential	845.805	1	24.1235	
Quiet sleep	**Weibull**	**990.749**	**2**	**0**	Shape 2.181 (95% CI: 1.897 to 2.507), SE: 0.155 Scale 34.243 (95% CI: 31.496 to 37.229), SE: 1.461
	Lognormal	999.533	2	8.7836	
	Exponential	1070.848	1	80.0989	

K, number of estimated parameters (degrees of freedom); Delta, the difference between each AIC and the smallest AIC; CI, confidence interval; SD, standard deviation; SE, standard error. The model highlighted in bold has the best goodness of fit.

### Active sleep bout durations were longest in preterm infants

Having established which distributions best fit sleep–wake bout durations, we assessed the influence of PMA (Figure 3 right panel). Wake bouts persisted for 15% longer with every week of PMA (exp[est] 1.146 [95% CI: 1.024 to 1.282]). Quiet sleep bouts persisted for 3% longer with every week of PMA (exp[est] 1.027 [95% CI: 0.997 to 1.058]). On the contrary, active sleep bouts were 6% *shorter* with every week of PMA (exp[est] 0.945 [95% CI: 0.896 to 0.997]). In all cases, the inclusion of PMA improved model fit (AIC wake: 261.185 vs. 264.629, quiet sleep: 989.493 vs. 990.749, and active sleep: 819.445 vs. 821.681). The inclusion of postnatal age or risk category did not improve PMA-only model fits. Please see Supplementary Information for further information on model fitting.

### Active sleep resilience to disturbance was highest in preterm infants

Prolonged sleep duration signifies high sleep pressure, i.e. need [44]. Having demonstrated that active sleep pressure was highest in very preterm infants, as indexed by its extended duration, we sought to confirm this in another way by testing whether their sleep was more resilient to disturbance. To do this, we examined whether awakenings from sleep were less likely to be evoked by a nociceptive or somatosensory perturbation in younger infants [45–48].

**Figure 4.**
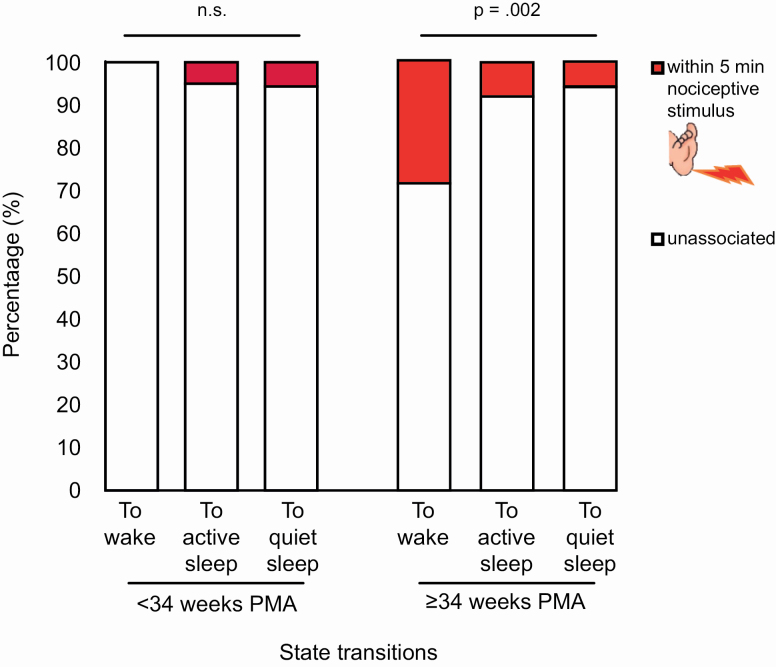
Transitions to wakefulness are associated with nociceptive stimuli in older infants (34–40 weeks PMA) but not younger infants (29–33 weeks PMA).

On the one hand, none of the eight awakenings in very and moderately preterm infants, which all emerged from active sleep, occurred within 5 minutes of a heel lance, demonstrating that their sleep was resilient to sensory perturbations. On the other hand, 10/35 (29%) awakenings in older infants clustered during the 5 minutes following a lance (skewness of latencies to wakefulness: 2.680), 9/10 of which emerged from active sleep. In contrast to awakenings, transitions from active to quiet sleep, or vice versa, did not cluster after a lance (only ≤8%: likelihood ratio test *p* = .002, Cramer’s V .248; Figure 4). This indicates that sleep in older infants is specifically vulnerable to awakenings following nociceptive stimuli, rather than more subtle changes in sleep depth. (These lance-associated awakenings did not last for significantly longer than other awakenings [inclusion of nociceptive perturbation variable did not improve wake bout durations model fit].)

In contrast to lances, there was no clustering of awakenings in the 5 minutes following a *non*-noxious stimulus in older infants (lesser skewness of latencies to wakefulness: 1.553; likelihood ratio test *p* = .720), indicating that only nociceptive stimuli evoked awakenings.

### Physiological stress lengthened wake bout durations

**Figure 5.**
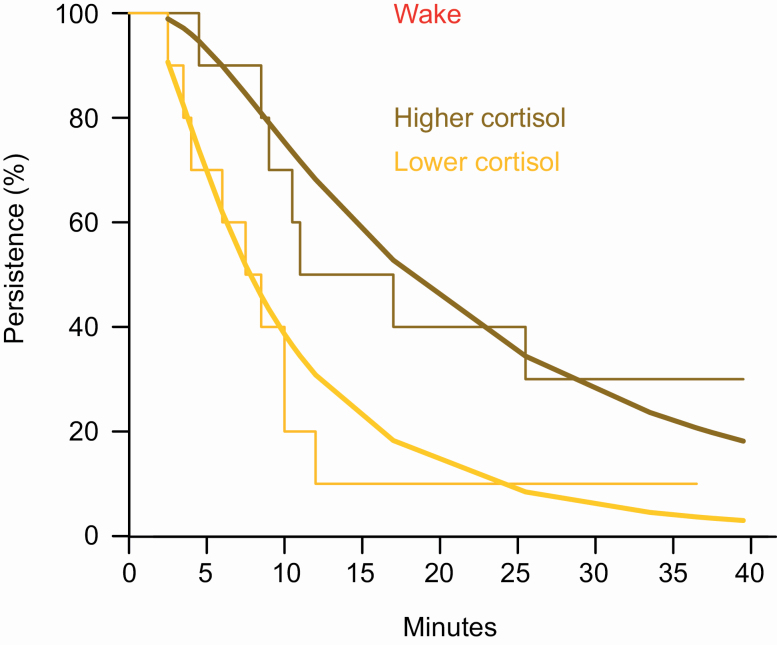
Fitted survival curves for wake persistence in infants with lower (yellow line) and higher (bronze line) physiological stress levels, alongside empirical Kaplan–Meier survival curves. Lower cortisol value range = .11 to .19 µg/dL, *n* = 10; higher cortisol value range = .21 to .81 µg/dL, *n* = 10. Confidence intervals omitted for clarity. The fitted curve is drawn with the covariate of PMA set to its mean value in the data.

Wake bouts persisted for longer with increasing cortisol level (exp[est] per µg/10 dL 1.335 [95% CI: 1.019 to 1.749]; Figure 5), and the addition of this variable improved PMA-only model fit (AIC 144.688 vs. 147.289; 20 wake onsets captured from 19 infants 32–40 weeks PMA, with offset captured in 16/20 instances). On the other hand, cortisol level did not improve sleep bout durations model fits.

### Long active sleep bouts were more likely to offset into quiet sleep than wakefulness

**Figure 6.**
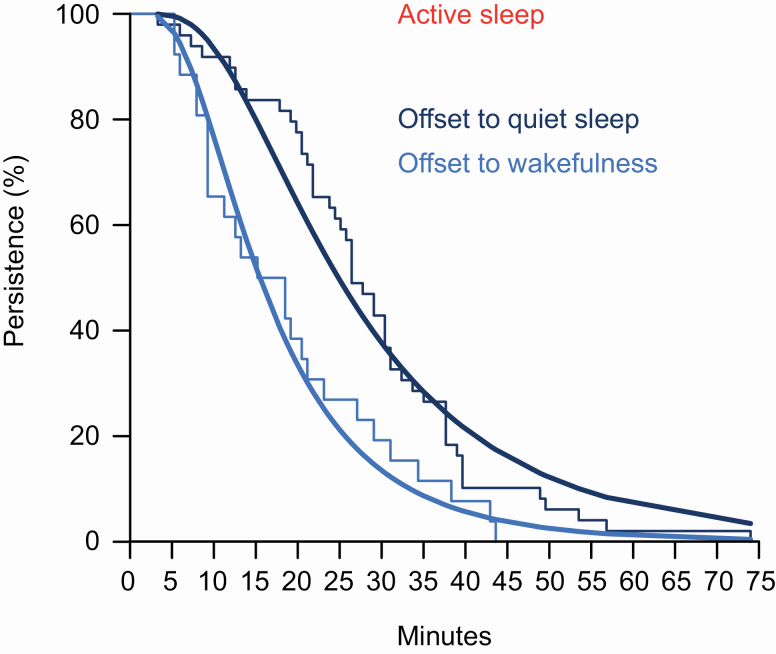
Fitted survival curves for active sleep persistence prior to the transition to quiet sleep (dark blue line) or wakefulness (mid blue line), alongside empirical Kaplan–Meier survival curves. Confidence intervals omitted for clarity. The fitted curve is drawn with the covariate of PMA set to its mean values in the data.

Next, we examined the influence of which transition occurred at bout offset. Active sleep could offset into either quiet sleep or wakefulness (unlike wakefulness and quiet sleep, which always offset into active sleep; Table 2). Active sleep bouts were 60% longer, which terminated in quiet sleep, when compared with bouts, which terminated in wakefulness (exp[est] 1.602 [95% CI: 1.203 to 2.133]; Figure 6). The addition of this variable improved PMA-only model fit (AIC 663.782 vs. 671.493).

## Discussion

Human fetuses are thought to remain almost exclusively asleep [49, 50] (intermittent motor activity is consistent with sleep during this developmental stage [9, 10]). Here, we show that preterm infants too can spend up to 97% of their time asleep, even following the physiological changes associated with birth [51, 52].

We demonstrate that the substructure of this sleep is uniquely regulated. Active sleep bout durations are prolonged and follow a heavy-tailed distribution. Quiet sleep bout durations follow a *light*-tailed distribution, concordant with bias toward the opposing (active) sleep state preventing extra-long quiet sleep bouts. This is the opposite pattern to older children and adults [53–57]. These sustained periods of active sleep may consolidate this state’s crucial developmental functions, including sensorimotor plasticity [9, 10, 58–60]. Meanwhile, the increasing probability of transition to quiet sleep with increasing active sleep length is consistent with a homeostatic process, which ensures quiet sleep can balance or complement these active sleep functions [61, 62]. For example, in adults, it has been theorized that REM sleep complements the accurate memory consolidation which occurs during non-REM sleep, by integrating these memories [63]. However, the order in which sleep states occur in infants, i.e. active sleep first [18, 64, 65], is the inverse of the pattern observed in adults [66]. This unique sleep behavior may point at differences in the functioning of the underlying circuitry, relative to adults. In adults, activity within the locus coeruleus of the pons can promote wakefulness, while cessation of its firing opens the gate to active sleep [67, 68]. Therefore, very low spontaneous firing within this structure, which has been reported in neonatal animals, may at least partly explain the dominance of active sleep in human infants and fetuses [69–71].

Although sleep pervades neonatal life, indeed, there were only 43 bouts of wakefulness across 175 infants, we demonstrate a developmental increase in wakefulness across the vulnerable preterm period, which occupied 15% of the time by full-term age. Increased firing in the locus coeruleus can promote a switch to wakefulness by exerting noradrenergic inhibition to the (otherwise sleep-promoting) ventrolateral preoptic region of the hypothalamus [68, 72] (Supplementary Figure S3). Therefore, age-related increases in locus coeruleus firing rate, and noradrenergic innervation of brain structures, may underlie the increase of wakefulness with maturation [70, 73]. We also show that awakenings can be triggered by nociception from late preterm age. This is consistent with neonatal animal models, which demonstrate that although the *basal* firing rate of locus coeruleus neurons is low, these neurons can fire robustly to nociceptive stimuli [64, 70, 74–79].

While nociception can trigger awakenings, physiological stress can *prolong* periods of wakefulness from at least as early as moderately preterm age, as in older children and adults [1, 40, 80, 81]. Cortisol is the final effector of the hypothalamic–pituitary–adrenocortical stress axis, and its production is mediated by the release of corticotropin-releasing hormone (CRH) in the hypothalamus. There is reciprocal stimulation between CRH release and locus coeruleus firing [82, 83], and between CRH release and the orexinergic neuronal activity, which reinforces locus coeruleus firing [84–90]. Therefore, stress could prolong wakefulness in human infants by influencing these positive feedback circuits, in keeping with neonatal animal models [91–93].

Taken together, infants who experience nociceptive stimuli and stress spend excessive time awake at the expense of sleep, during a sensitive period in which sleep supports cortical development [94, 95], but this additional wakefulness may be of relatively little value [3–5]. For example, vigilance to a nociceptive stressor could be less useful when infants do not have the same capacity to “fight or flight” as an adult. Further, in this cohort, one advantage of wakefulness—being picked up by a caregiver—did not apply until full-term age (Supplementary Information and Supplementary Figure S4).

Sleep–wake architecture in human infants has been associated with cognitive and sensorimotor outcomes [96–99]. However, environmental interventions to improve sleep quality have demonstrated only modest—and often discrepant—results [100–103], in part, because of an incomplete understanding of sleep–wake regulation in this population. Here, we address this by formulating a conceptual model consistent with basic neurobiology, which could support the simulation and preclinical planning of hypothesis-driven interventions. In particular, we identified two tractable factors—noxious procedures and physiological stress—which could be targeted to protect sleep in this vulnerable population. For example, it may be advantageous to avoid periods of active sleep, which are vulnerable to nociception-evoked awakenings, when conducting necessary noxious procedures. Given that active sleep is associated with specific cardio-respiratory markers (Figure 1), it would be feasible to integrate a state-detection algorithm into the cot-side heart- and respiratory-rate monitors, which would flag this state to staff. Secondly, as we show here that stress curtails sleep by extending wakefulness, future studies should assess whether sensory interventions—which can reduce physiological stress in infants [104, 105]—could thereby promote sleep. Particular emphasis should be paid to the effectiveness of interventions, which are available 24/7, i.e. even when parents are not present, such as supportive positioning in the cot/incubator [106]. Quiet sleep shows excellent promise as a post-intervention outcome measure, as it has high inter-bout consistency that makes the identification of outliers straightforward, can be parsimoniously modeled with a single parameter (PMA) [107], and is associated with neurodevelopmental outcome [99].

This work has some limitations. There are a few reports that sleep duration is a relatively insensitive index of sleep pressure early in development [45, 108]. However, our interpretation is strengthened by the converging evidence that preterm infants’ sleep is also resilient to nociceptive perturbations, a separate index of sleep pressure validated by experiments in neonatal nonhuman primates, rats, and mice [45–48]. A second limitation is that the recording length was insufficient to capture multiple sleep–wake cycles. Although it has been reported that short-term neonatal sleep–wake recordings are representative of long-term recordings [16], future work should aim to replicate our results using 24 hours, and ideally serial, recordings to capture longitudinal trajectories. Thirdly, the analyses regarding physiological stress and nociceptive procedures were conducted on relatively small samples, given the complexity of combining and timing multiple physiological measures during the same recording.

## Conclusions

Sleep–wake regulation evolves with maturity from the equivalent of the late fetal period to adulthood. Here, we show that preterm infants have extraordinarily high active sleep pressure. With increasing age, wake bouts become longer, and nociception and stress additionally funnel infants toward this state of heightened vigilance, which will eventually occupy two-thirds of adult life, an enormous increase from the 3% occupancy in very preterm infants.

## Supplementary Material

zsaa148_suppl_Supplementary_Material
