# Supplementary material for: Sleep–wake regulation in preterm and term infants
Source: Sleep. 2020 Aug 8;44(1):zsaa148. doi: 10.1093/sleep/zsaa148 (PMC7819838; doi:10.1093/sleep/zsaa148)
Supplement: zsaa148_suppl_Supplementary_Material [file zsaa148_suppl_supplementary_material.docx]

**Sleep-wake regulation in pre-term and term infants**

Anastasis Georgoulas ^a*^, Laura Jones ^b*^, Maria Pureza Laudiano-Dray ^b^, Judith Meek ^c^, Lorenzo Fabrizi ^b^, Kimberley Whitehead ^b^

^*^ Contributed equally as co-first authors.

**Affiliations:** ^a^ Research IT Services, University College London, London, United Kingdom; and ^b^ Department of Neuroscience, Physiology and Pharmacology, University College London, London, United Kingdom; and ^c^ Elizabeth Garrett Anderson Wing, University College London Hospitals, London, United Kingdom

**Address correspondence to:** Kimberley Whitehead, G17 Medawar Building, University College London, Gower Street, London, WC1E 6BT, [[k.whitehead@ucl.ac.uk](mailto:k.whitehead@ucl.ac.uk)], +44(0)20 7679 3533

**Supplementary Information Text**

**Infants at higher risk of adverse neurodevelopment**

Twenty-six of 175 subjects were categorised as being at higher risk of adverse neurodevelopment (beyond that associated with degree of prematurity). Reasons for being categorised as higher risk included: chromosomal abnormality associated with intellectual disability, brain parenchymal tissue loss, severe intra-uterine growth restriction (defined here as abnormal antenatal Doppler ultrasound measurements, delivered early secondary to concerns about foetal growth, or ≤0.4th birth weight centile), symptomatic congenital heart disease, or referred - for any other reason - for formal neurodevelopmental or neurological assessment.

**Further information on model fitting**

If quiet or active sleep terminated with transitional sleep (e.g. Fig. S2 upper panel), these transitional sleep epochs were not included within the state’s calculated duration, as this undermined model fit (higher AIC: quiet sleep 991.071 vs. 989.493 (10/157 bouts); active sleep 820.664 vs. 819.445 (3/183 bouts)).

This is supportive that, when transitional sleep is scored conservatively (i.e. resulting in mean recording time proportions of a few percent, as here (Fig. 2) ^1^), this state is independent of quiet and active sleep.

**Caregiver holding**

Awakenings in full-term infants evoked an increase in the proportion of infants held (peak approximately 5 minutes post-awakening), but not in pre-term infants (proportion held 0 vs. 5 minutes post-awakening remained associated in pre-term infants (Fisher’s Exact Test of association p <.001), but dissociated in full-term infants (p = 1.000). Analysis data comprised 39 awakenings after which the recording lasted for ≥5 minutes: 25 pre-term (mean 34+1 weeks+days postmenstrual age (PMA), 14 full-term (mean 38+3 weeks+days PMA); Fig. S4).


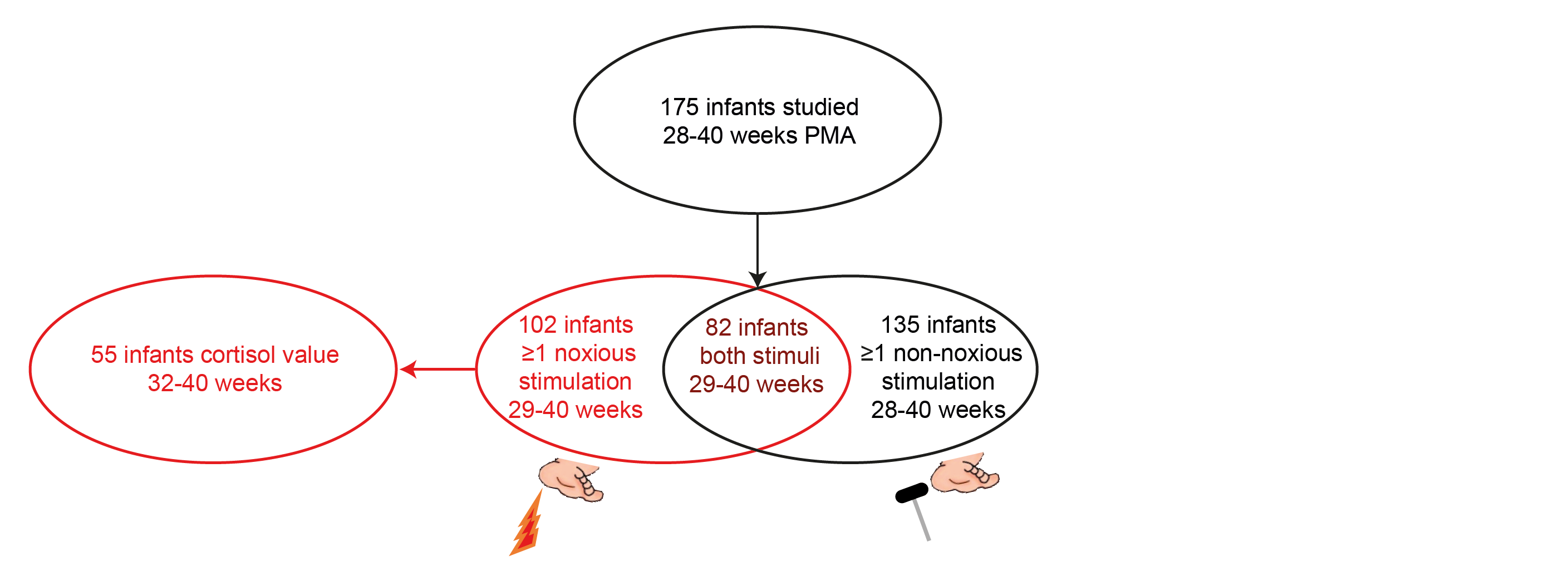


**Figure S1.** Postmenstrual age (PMA) distribution across flowchart of Experimental Protocol.


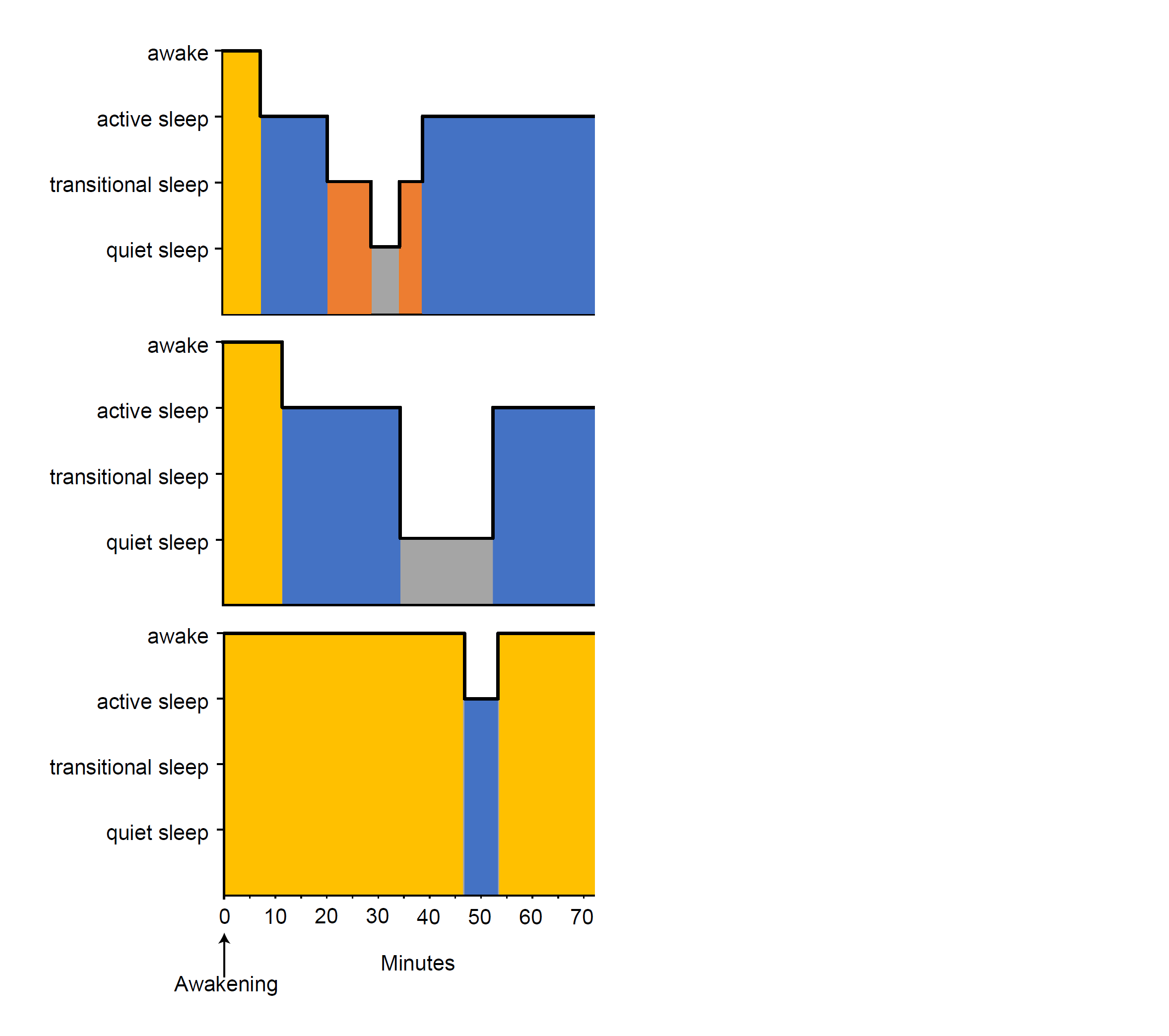


**Figure S2.** Three examples of sleep-wake time courses in infants of postmenstrual age 30+3, 32+6, and 35+4 weeks+days (from top to bottom). Each time course has been plotted from an awakening from active sleep (0 minutes). Note the divergence in time courses, from this common starting point.


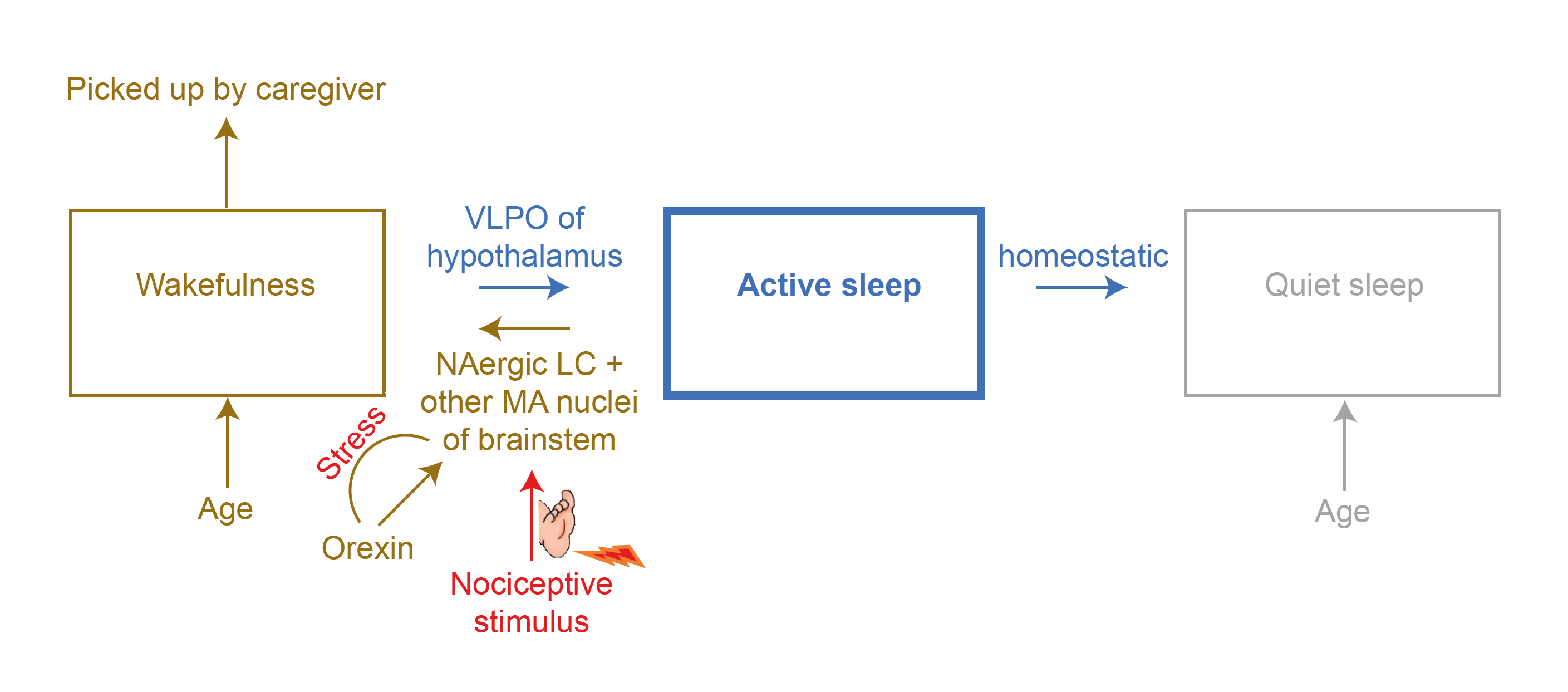


**Figure S3.** Summary of the current evidence (including the present study) about the regulation of sleep-wake bouts in infants. VLPO = ventrolateral preoptic region. NA = Noradrenergic. MA = Monoaminergic.


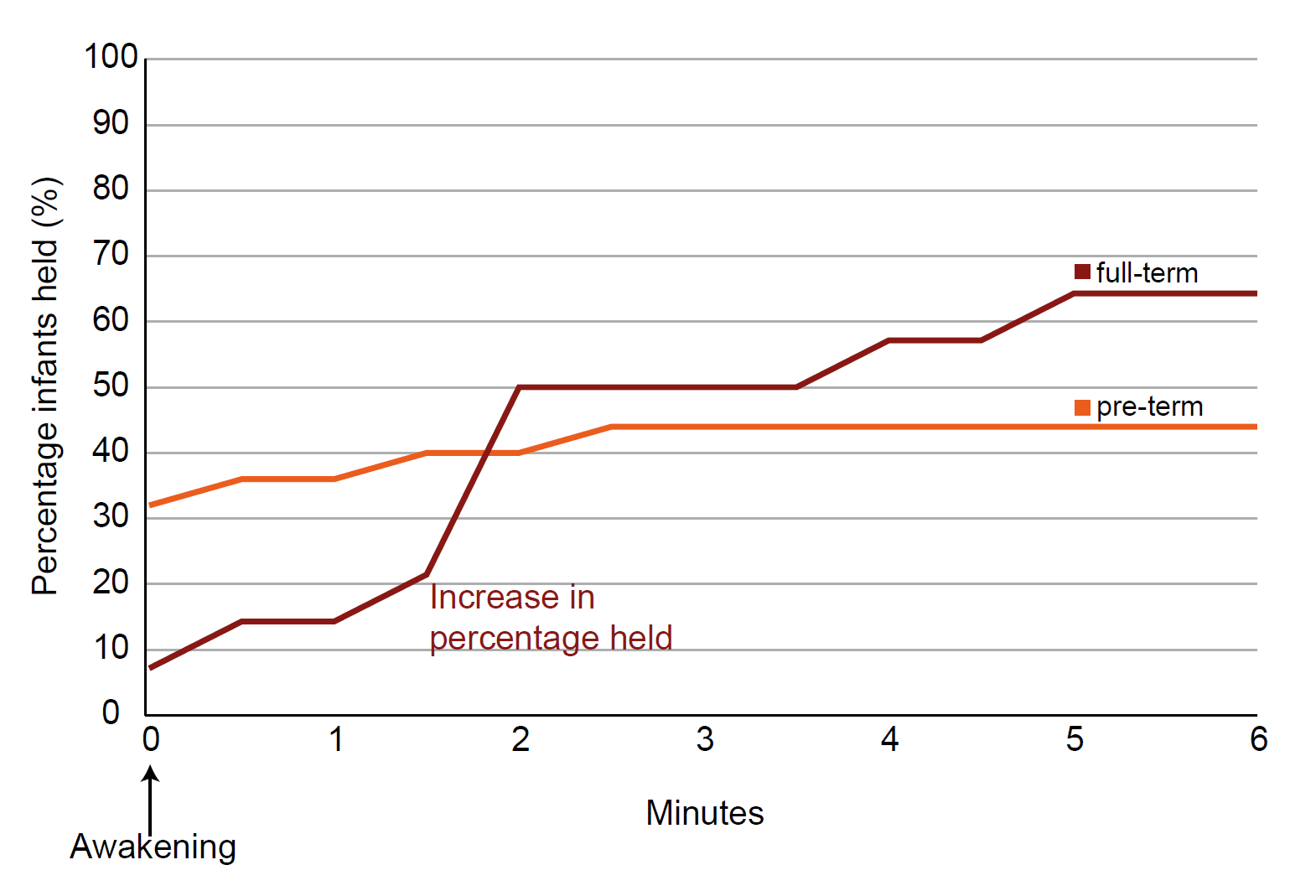


**Figure S4.** Full-term infants are more likely to be picked up by a caregiver upon awakening.

**References**

1. Korotchikova I, Connolly S, Ryan CA, et al. EEG in the healthy term newborn within 12 hours of birth. *Clinical Neurophysiology*. 2009;120(6):1046-1053. doi:10.1016/j.clinph.2009.03.015
